# ESGE Journal: Time for a new start

**Published:** 2019-06

**Authors:** Grigoris Grimbizis

The year 2019 will probably remain a semantic cornerstone in the evolution of the European Society for Gynaecological Endoscopy (ESGE). A new era is beginning in the scientific face of our Society with its new official journal Facts, Views & Vision in ObGyn (FVVO), fulfilling the vision of our pioneers. Since its foundation in 1994, our Society has aimed to encourage the exchange of clinical experience, scientific thought and investigation among European gynaecological endoscopists and practitioners in related techniques.

It was obvious from past experience that the official journal of our Society has to provide broad visibility of the published material: our new journal, being open access, is freely accessible to all readers. But not only that; with its 10 years successful history, FVVO succeeded to be included in Pubmed. Thus, the scientific content of the journal is searchable in the main scientific database used by those interested in being informed on a topic of our field.

The open access mode and the inclusion in Pubmed are both crucial for the official journal of our Society and ESGE members as the research published in FVVO is visible to the wider scientific community and ultimately will be cited.

A clear advantage of the new official journal is that it combines the electronic open access mode with a printed version: this was a longstanding wish of many of our members. The printed version of the journal is really unique as the artist Koen Vanmechelen designs it: the front page is always an art work and selected papers are also covered by special art pieces. Furthermore, the ESGE members and the members of the ESGE Corporate Member Societies will have the opportunity to have the printed version only at the mailing cost.

Being an open access journal, it is usual that the authors have to pay for their publication. This is indeed the case for the non-ESGE members. But the ESGE members and the members of the Corporate Member Societies can publish their work, following peer reviewing, free of charge: the Society will cover the expenses of their publication.

However, most importantly, our new official journal will be owned by ESGE. This will provide a steady environment for the scientific work in our field and will also give the opportunity to our Corporate Member Societies to be expressed officially by a journal. It is noteworthy that “The Walking Egg” will continue to be present in the journal and that the International Society for Gynaecological Endoscopy (ISGE) has selected FVVO as its official journal.

We would like to underline that our intention is to combine the heritage from the successful history of FVVO with the ESGE scientific production and reputation. Therefore, the current Editor in Chief, Prof. Willem Ombelet will continue his successful leadership, supplemented by the ESGE Editor Prof. Ertan Saridogan.

We encourage the whole ESGE community to support this new era in the scientific evolution of our Society.

Grigoris Grimbizis ESGE President

